# Efficacy of Cyproheptadine Monotherapy in Hepatocellular Carcinoma With Bone Metastasis: A Case Report

**DOI:** 10.3389/fonc.2021.620212

**Published:** 2021-10-20

**Authors:** Yu-Min Feng, Tsung-Hsien Chen, Dara Berman, Chu-Kuang Chou, Kai-Sheng Liao, Ming-Chih Hsieh, Chi-Yi Chen

**Affiliations:** ^1^ Divisions of Gastroenterology and Hepatology, Department of Internal Medicine, Ditmanson Medical Foundation Chiayi Christian Hospital, Chiayi, Taiwan; ^2^ Department of Internal Medicine, Ditmanson Medical Foundation Chiayi Christian Hospital, Chiayi, Taiwan; ^3^ Charles H. Dyson School of Applied Economics and Management, SC Johnson College of Business, Cornell University, Ithaca, NY, United States; ^4^ Clinical Trial Center, Ditmanson Medical Foundation Chiayi Christian Hospital, Chiayi, Taiwan; ^5^ Department of Nursing, Chung-Jen Junior College of Nursing, Health Sciences and Management, Chiayi, Taiwan; ^6^ Department of Pathology, Ditmanson Medical Foundation Chiayi Christian Hospital, Chiayi, Taiwan; ^7^ Department of Radiology, Ditmanson Medical Foundation Chiayi Christian Hospital, Chiayi, Taiwan

**Keywords:** cyproheptadine, hepatocellular carcinoma, monotherapy, alpha fetal protein, case report

## Abstract

**Background:**

Hepatocellular carcinoma (HCC) is one of the most common causes of cancer-related deaths worldwide. Particularly, cases of bone metastasis have poorer prognoses.

**Case Presentation:**

A 62-year-old woman with suspected advanced HCC accompanied by bone metastasis with severe back pain and sciatica showed disease remission after cyproheptadine monotherapy. Initially, her serum alpha fetal protein (AFP) level was high, reaching up to 17697.62 ng/ml. A dose of 4 mg cyproheptadine, 3 times a day for 17 months was prescribed as the only treatment. Within 3 months, the serum AFP level gradually normalized down to 4.3 ng/ml. Both liver biopsy and bone biopsies were subsequently performed after 2 weeks of cyproheptadine. The results showed no malignancy. During the 34 months of follow-ups, the serum AFP remained normal in the range of 1.05 to 2.86 ng/ml. The patient has survived for 5 years without back pain and sciatica thus far.

**Conclusions:**

This is the first report to investigate a successful clinical approach in cyproheptadine monotherapy for an advanced HCC patient with bone metastasis. We recommend cyproheptadine as a potential anti-HCC agent for the treatment of HCC with bone metastasis, but more related studies such as prospectively clinical trials, and ideally randomized trials are still needed.

## Introduction

Liver cancer is one of the most common causes of cancer death. Hepatocellular carcinoma (HCC) is the sixth most common cancer and the fourth most common cause of cancer-related death (1). However, HCC is often diagnosed during its advanced stages. The lung, abdominal lymph nodes, and bone are the most common sites of extrahepatic metastasis (2, 3). Bone metastasis typically involves the spine, pelvis, and ribs. For patients with HCC metastasis, systemic therapies using small molecule drugs, that target various signaling pathways, have been applied. Sorafenib was the first systemic targeted drug approved by the FDA for the treatment of advanced HCC (4). There have been some other kinase inhibitors and immune checkpoint inhibitors emerging in recent years, but all of them are very expensive with some severe adverse events (5, 6). Otherwise, they achieve complete response in small proportion. Cyproheptadine may have some role in treating some HCC patients.

Cyproheptadine is a first-generation antihistamine. It is also a serotonin antagonist with anticholinergic and sedative effects. Currently, cyproheptadine is used clinically to stimulate weight gain and treat various health problems such as atopic dermatitis, anorexia, and migraines. Additionally, it was reported to be included in a novel drug combination therapy (cyproheptadine combined with thalidomide or sorafenib) for treating HCC patients with lung metastasis, and advanced HCC (7, 8). Bone metastases from HCC result in extremely poor prognoses, with a median survival of only 1 to 2 months (9). Here, we describe a case of an HCC patient with bone metastasis who attained remission after cyproheptadine monotherapy.

## Case Report

**Figure 1:**
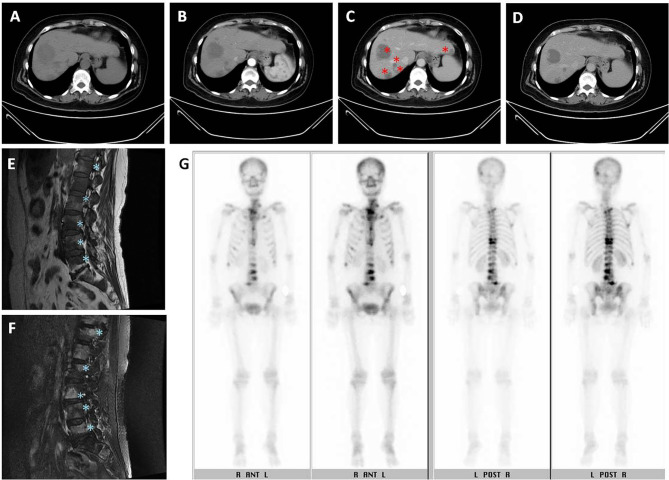
Initial imaging examination. **(A–D)** Dynamic abdominal computed tomography (CT): **(A)** multiple variable sized tumor lesions in both hepatic lobes with hypodensity in precontrast image, **(B)** persistent poor enhancement showed in arterial phase, **(C)** portal venous phase taken on November 26, 2015, original tumor lesions indicated by red asterisk symbol, and **(D)** delayed-postcontrast phase. **(E, F)** L-spine MRI showed multiple tumor lesions indicated by blue asterisk symbol. **(E)** Fluid attenuated inversion recovery (FLAIR) T1-weighted MRI. **(F)** Fast relaxation fast spin echo (FRFSE) T2-weighted MRI. **(G)** Tc-99m MDP whole body bone scan showed multiple increased tracer uptake in the skeletal system, compatible with multiple bone metastases.

On October 27, 2015, a 62-year-old woman visited the neurosurgery department with main complaints of persistent severe low back pain and sciatica for more than one month. She was unable to walk due to lower back pain and sciatica in both lower limbs. The patient was referred to our hospital for further examinations, and dynamic abdominal computed tomography (CT) taken on November 26, 2015 suggested multiple variable sized tumor lesions in both hepatic lobes. Poorly differentiated HCC or cholangiocarcinoma should be suspected (**Figures 1A–D**). The spinal magnetic resonance imaging (MRI) scan suggested multiple bony lesions and favored bony metastasis (**Figures 1E, F**). She had chronic hepatitis C-related liver cirrhosis for years. The blood test revealed elevated alpha-fetoprotein (AFP) level at 17697.62 ng/ml, high above the normal range of 0-8.78ng/ml. She was referred to our outpatient department of gastroenterology on November 18, 2015. Since her symptoms were serious and we had studied the effect of cyproheptadine in the treatment of HCC in the past (3, 7), we prescribed cyproheptadine (a dose of 4 mg thrice daily) (3), and silymarin (a dose of 150 mg thrice daily) for two weeks right away. Thereafter, the same doses were continued daily until April 6, 2017. Since silymarin is a vitamin supplement to boost liver health, we consider the prescription of cyproheptadine as the only medical treatment we perform.

On November 28, 2015, she was admitted to the hospital. On December 7, 2015, laboratory examination revealed a white blood cell count of 2,290/ul; hemoglobin 9.2 g/dl; platelet count 65,000/ul; aspartate aminotransferase 49 U/l; alanine aminotransferase 43U/l; albumin 3.7 g/dl; total bilirubin 0.34 mg/dl; and prothrombin time 12.7 sec (**Table 1**). Both liver ultrasound and CT revealed multiple liver tumors. A whole-body bone scan revealed multiple sites of increased tracer uptake in the skeletal system and suspicious bone metastases (**Figure 1G**).

**Table 1:** Patient’s initial laboratory values and normal ranges.

Variable	Laboratory Values	normal range
Hemoglobin (g/dl)	9.2	12-16
White-cell count (per ul)	2,290	3500-9900
Platelet count (per ul)	65,000	130,000-400,000
Aspartate aminotransferase (GOT) (U/L)	49	8-38
Alanine aminotransferase (GPT) (U/L)	43	4-44
Albumin (g/dl)	3.7	3.81-5.31
Total bilirubin(mg/dL)	0.34	0.2-1.2
Prothrombin time (sec)	12.7	9.4-12.5

**Figure 2:**
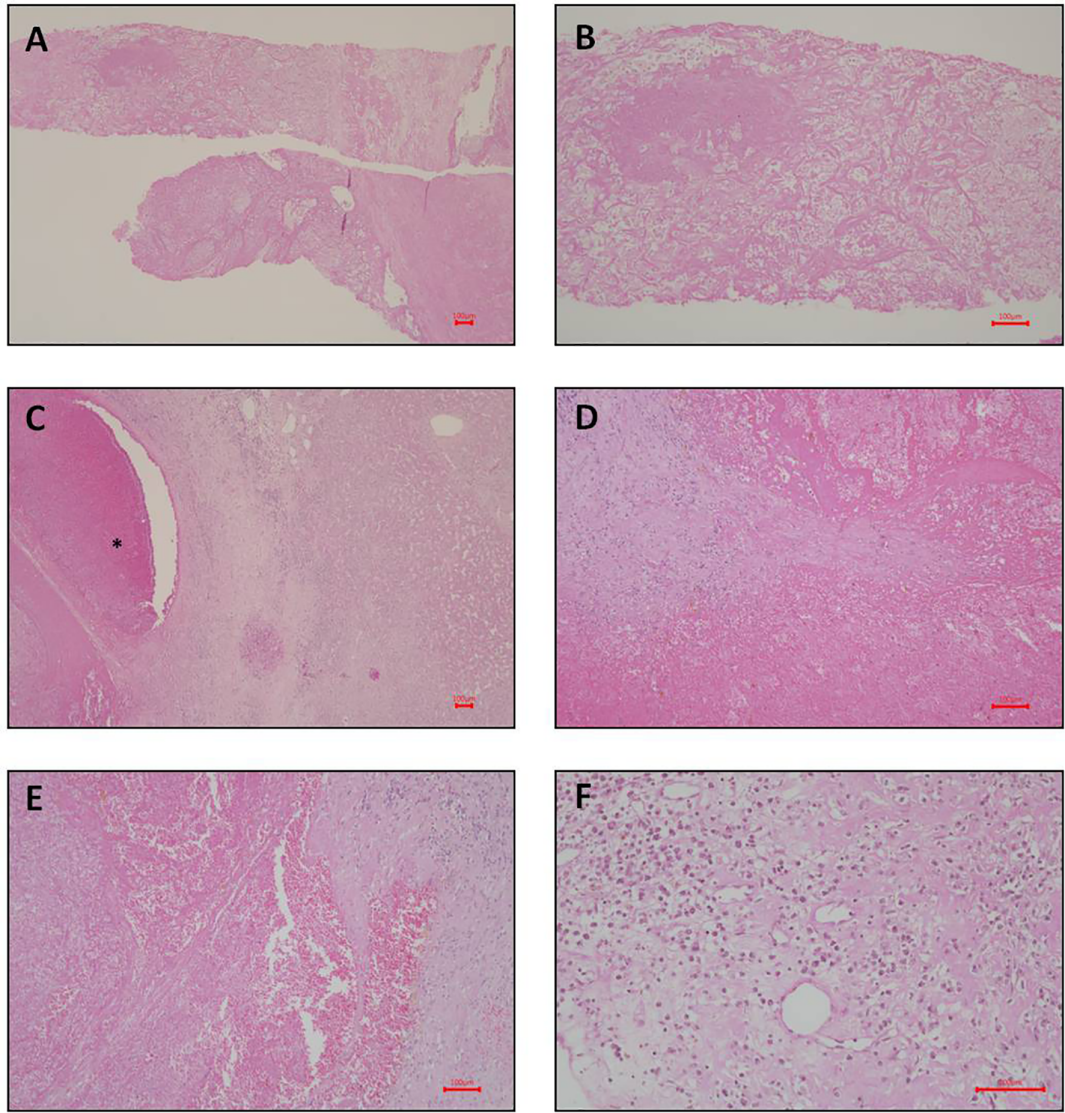
Photomicrograph of liver pathology. (**A–C**: liver biopsy; **D–F**: liver pathology after left partial segmentectomy). **(A)** Necrosis and fibrin shown on liver biopsy. Hematoxylin and eosin stain; magnification, 40X. **(B)** Higher magnification of necrosis and fibrin shown on liver biopsy. Hematoxylin and eosin stain; magnification, 100X. **(C)** Organizing hematoma (indicated by asterisk symbol in the photomicrograph) shown on the left side. The normal liver parenchyma shown on the right side. Hematoxylin and eosin stain; magnification, 40X. **(D)** Higher magnification of the hematoma. No viable cell can be identified. Hematoxylin and eosin stain; magnification, 100X. **(E)** Another view of the hematoma. Hematoxylin and eosin stain; magnification, 100X. **(F)** Focal chronic inflammation and fibrosis shown on liver. Hematoxylin and eosin stain; magnification, 200X. Scale bar = 100 microns.

On November 30, 2015, she underwent a liver core biopsy, which showed necrosis, but no cancer cells (**Figures 2A, B**). On December 10, she underwent resection of one suspected liver tumor in the left lobe. The pathological report documented organizing hematoma with fibrosis, and no evidence of malignancy was found (**Figures 2C–E**). Needle biopsy was performed during this operation for tumors of the right hepatic lobe. The pathological report showed patchy foci of necrosis with marked fibrosis and chronic inflammation (**Figure 2F**). The serum AFP level decreased to 324.08 ng/ml on December 23, 2015.

**Figure 3:**
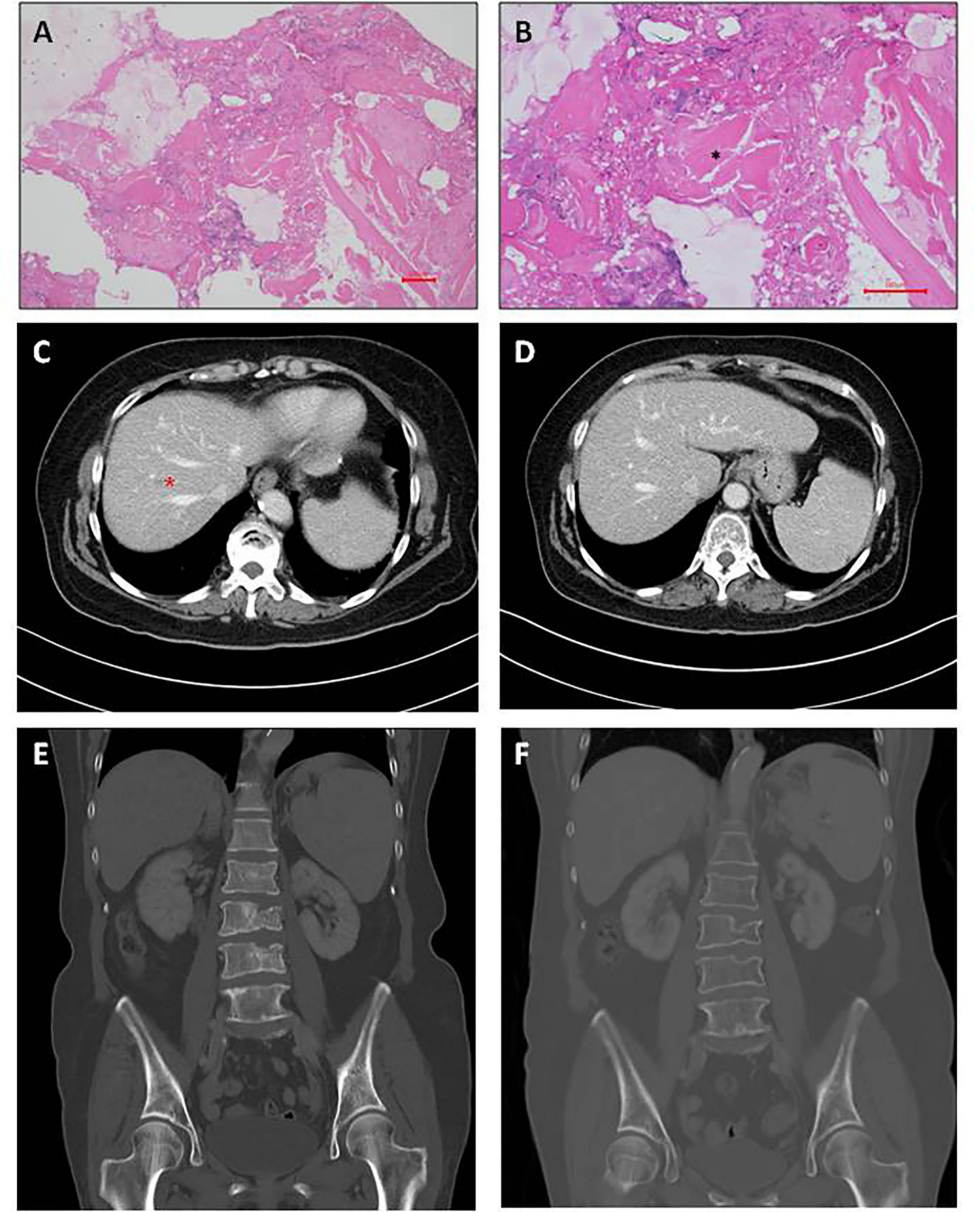
Post treatment imaging findings **(A)** Photomicrograph of spine pathology. Focal necrosis shown on L3 spine biopsy. No evidence of malignancy can be found. Hematoxylin and eosin stain; magnification, 100X. **(B)** Necrotic bone (indicated by black asterisk symbol in the photomicrograph) shown on spine biopsy. Hematoxylin and eosin stain; magnification, 200X. **(C)** Dynamic abdominal CT portal venous phase taken on February 9, 2017. Original tumor lesions indicated by red asterisk symbol. **(D)** Dynamic abdominal CT portal venous phase taken on December 12, 2018. **(E)** Initial coronal CT of L-spine taken on November 26, 2015. **(F)** The followed coronal CT of L-spine taken on December 12, 2018.

Since liver biopsies were done thrice but did not achieve definite results, lumbar spine surgery was performed on December 26, 2015. Bone biopsies of the second and third lumbar vertebrae were performed, and the results were L2 biopsy: fibrosis, L3 biopsy: focal necrosis and new bone formation (**Figures 3A, B**). The serum AFP level gradually decreased down to 182.72 ng/ml on December 28, 2015, 52.04 ng/ml on January 8, 2016, and 10.23 ng/ml on January 26, 2016. After multiple biopsies without a definite result, the patient claimed to have become well. She was able to walk normally. Her back pain and sciatica disappeared. After she was discharged from the hospital on December 31, 2015, her AFP level was monitored in the clinic, and it decreased to 4.3 ng/ml on February 11, 2016. Thereafter, it remained the normal range until now. Although the AFP level became normal, hepatitis C viral (HCV) RNA level was detected up to 868561 IU/ml on April 6, 2017. The genotyping is 1b. The patient was treated with direct-acting antiviral agents (DAA) for hepatitis C for 12 weeks from May 19, 2017 to August 11, 2017. The DAA regimens include ombitasvir 25 mg/day, paritaprevir 150mg/day, ritonavir 100mg/day and dasabuvir 250 mg twice daily. Sustained virologic response was achieved on November 3, 2017. Follow-up CTs were performed on February 9, 2017 (**Figure 3C**) and December 12, 2018 (**Figure 3D**), respectively. Results showed little tumor (**Figure 3C**) and then no tumor (**Figure 3D**), respectively, after treatment. They contrasted with the initial one (**Figure 1C**) taken on November 26, 2015, which showed that she had several lesions in both hepatic lobes. We used hepatic veins as a landmark to verify that the pictures showing little lesions (**Figure 3C**) and no lesions (**Figure 3D**) are in the same section as the initial one showing multiple lesions (**Figure 1C**). Remarkable treatment effect was noted. Initial coronal CT of L-spine (**Figure 3E**) showed multiple mixed osteoblastic and lytic change in L-spine bony structure, with pathological fracture of L3-4, suggestive bony metastasis. The followed one (**Figure 3F**) revealed focal lytic change of L-spine and no progression of compression fracture, nor more trabecular destruction of L-spine vertebral body.

**Figure 4:**
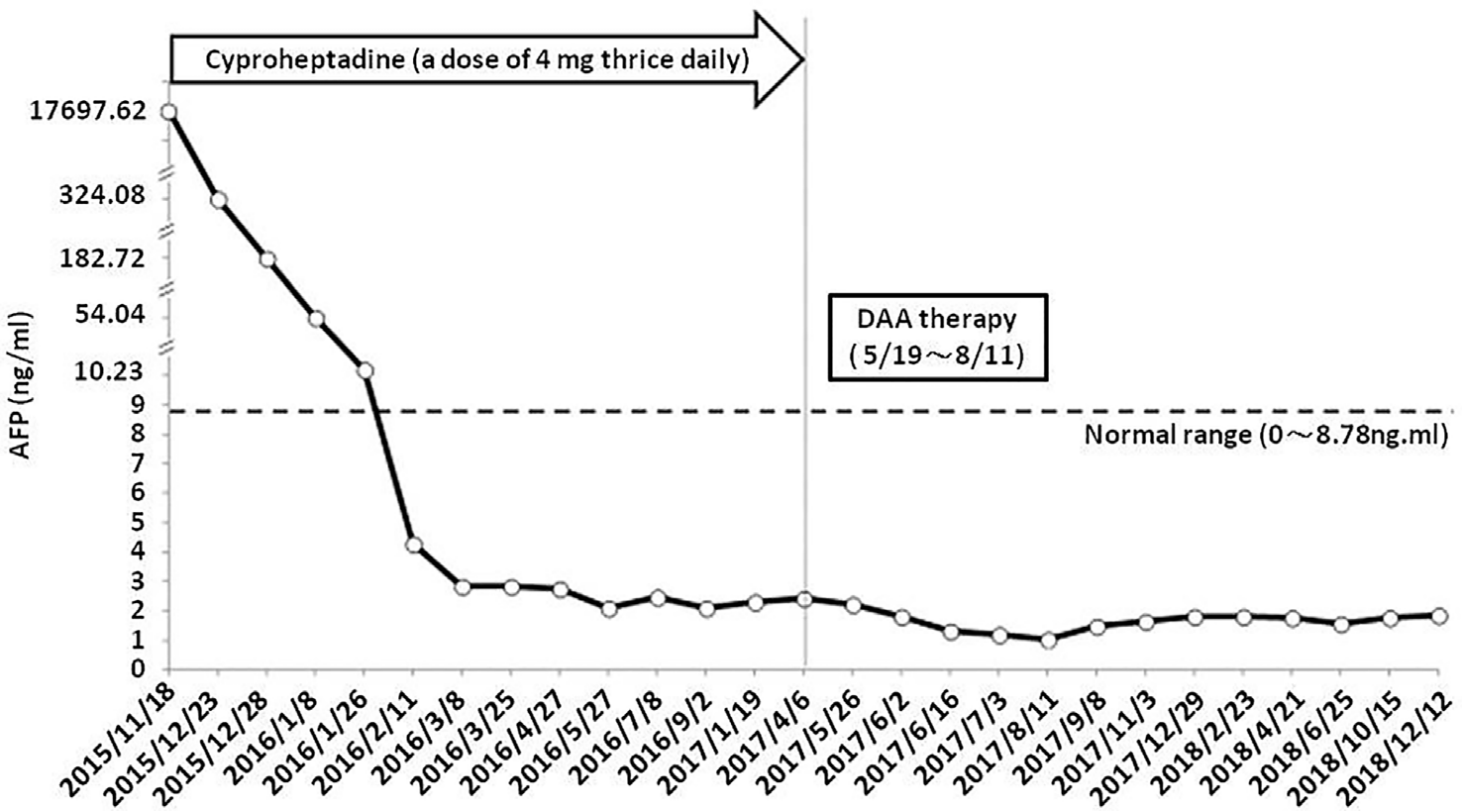
Timeline of treatment process and patient’s corresponding serum AFP levels. DAA, direct-acting antiviral agents.

It was clinically determined that her liver tumor, complicated by bone metastasis and nerve compression, had completely regressed. **Figure 4** illustrates the timeline of the complete treatment procedure and the corresponding serum AFP levels. The patient has remained alive for five years without back pain and sciatica thus far.

## Discussion

To the best of our knowledge, this is the first case report describing the successful and efficacious use of cyproheptadine monotherapy on a patient in the advanced stage of HCC. The use may significantly improve the therapeutic approach in treating HCC patients with bone metastasis.

Why did we think that this is an HCC patient? The results from biopsies of liver and bone did not show definite cancer cells. The diagnosis of HCC without pathological correlation was achieved by analyzing serum α-fetoprotein levels combined with imaging techniques. The diagnosis was based mainly on her clinical manifestations as well as the high level of AFP (i.e. 17697.62 ng/m). She had chronic hepatitis C related cirrhosis with a high risk for the growth of HCC. She had multiple space-occupying solid tumors in both liver and bone. She had obvious symptoms and signs of spinal metastasis (low back pain and sciatica), which subsided when her serum AFP level gradually decreased to the normal range.

High levels of serum AFP can be a sign of HCC or cancer of the ovaries or testicles (endodermal sinus tumor), as well as noncancerous liver diseases such as cirrhosis and hepatitis. She had no cancer of ovaries found in a whole abdomen CT scan. When the value of serum AFP is greater than 400 ng/ml, it is 75%-91% specifically considered diagnostic for HCC in the proper clinical context, including suitable radiologic findings and if it is followed by space-occupying solid lesion(s) in cirrhotic liver (10). We must consider this given that diffusely growing HCC may be difficult to be interpreted through imaging. Though pathological reports did not prove it to be a case of HCC clearly, based on her conditions and other tests, from a clinical point of view, it would be undoubtedly diagnosed as an advanced case of HCC.

How did we explain the pathological results? We assumed that the cancer cells had large areas of necrosis before biopsy. The patient has started to take cyproheptadine since November 18, 2015, two weeks before her first liver biopsy, which was performed on November 30, 2015. Our assumption was based on our previous experience in definite HCC patients with lung metastasis (3). After the combined use of thalidomide and cyproheptadine on December 2, 2011, the serum AFP level of the patient decreased from 122ng/ml on November 21, 2011, to 5.5 ng/ml on January 13, 2012. Images of CT scans taken on January 19, 2012 confirmed the disappearance of bilateral lung.

In this case, we prescribed only cyproheptadine based on our prior analysis of its anti-cancer properties (3, 7); it may stimulate the immune system; it may suppress prolactin, growth hormone and cortisol; thalidomide was not added at that time due to precautions of its possible adverse side effects. One tablet of cyproheptadine is 4 mg; A dose of 4 mg thrice daily is commonly recommended for colds and allergies, and it demonstrates being safe yet effective on HCC patients based on our previous report (3). Because the patient responded well to the single drug therapy while we were waiting for the results of biopsies, we decided to continue it. Because the patient’s immune system responded well to the single drug therapy while we were waiting for the results of biopsies, we decided to continue it. After three months, the results of her blood test (e.g. AFP level) and CT scan (e.g. tumor) all returned normal ranges. However, the results of CT scan still showed traces of suspected tumors. To avoid recurrence, we continued the same therapy for another fourteen months until there were no more traces of suspicious tumors, tests and symptoms.

The role of cyproheptadine in HCC patients with metastasis has accumulated interest in both the biological and clinical fields. This case documents a suspected advanced HCC patient with bone metastasis being cured with cyproheptadine alone. Previous research reports mainly used cyproheptadine as part of a combined chemotherapy regimen to treat HCC. A previous case report that observed two patients with liver cancer complicated by lung metastasis demonstrated that treatment with thalidomide (50 mg twice daily) and cyproheptadine (4 mg thrice daily) for several months completely eliminated their tumors (3). Another study revealed that both overall survival and progression-free survival of patients with advanced HCC, treated with both sorafenib and cyproheptadine, were significantly longer than those of the control group (i.e. sorafenib alone). The differences were 11.0 months vs. 4.8 months; 7.5 months vs. 1.7 months (7). A large cohort of stratified statistical data published in 2017 (using the database of 70,885 HCC patients from Taiwan Cancer Registry Database) showed that among the patients who received CY monotherapy, the risk of all-cause deaths from stages I to IV was reduced (all *P* < 0.0001 and adjusted hazard ratio 0.61, 0.57, 0.54, and 0.52 for stages I, II, III, and IV, respectively (8). It did not exhibit the clinical outlook of related cases because patients’ initial laboratory values and complete treatment procedures are restricted from the cancer registry database. In this case report, we recorded the actual dose, dose regimen, test results and the complete treatment procedure. It also reinforces the results of previous cohort study (8).

We are certain that this is not a case of spontaneous regression of HCC. The patient did not receive other type of anti-cancer therapy before or after cyproheptadine monotherapy; her symptoms vanished, and the HCC had nearly complete remission while she was treated continuously with cyproheptadine monotherapy.

Cyproheptadine has cytotoxic effects and increased annexin V staining for cell death, which raise the levels of poly ADP-ribose polymerase (PARP) and its cleaved form, inducing apoptosis in HCC cell lines (3, 7, 11). In addition, cyproheptadine can suppress the PI3K/AKT signaling pathway in carcinoma cells and multiple myeloma cells, leading to cell cycle arrest in the G1 phase and G1/S transition, resulting in apoptosis and necrosis (7, 12). Apoptosis or programmed cell death is one of the primary mechanisms by which multi-cellular organisms control normal development and prevent aberrant cell growth (13). Thus, cyproheptadine could regulate the relevant pathways, inhibit tumor development, and accelerate apoptosis of tumor cells.

Additionally, in the liver inflammatory process, the hepatocytes die more frequently due to programmed cell death. However, chronic liver inflammation may lead to an increase in cell compensatory proliferation, thereby promoting the development of liver cancer (14). Chronically high levels of circulating inflammatory promoters, such as serotonin and histamine, can damage the liver (15). Serotonin may facilitate HCC growth, aggravate viral hepatitis, and play a crucial role in the progression of hepatic fibrosis (16). Serotonin is also involved in cancer cell migration, metastasis and angiogenesis. However, cyproheptadine is a serotonin antagonist and antihistamine agent, and the anti-inflammatory activity is presumably due to its anti-serotonin effect. Cyproheptadine alleviates the stress and inflammation in the liver, allowing it to better perform its detoxification functions.

Cyproheptadine was discovered to be able to treat not only HCC, but bladder cancer as well (17). There was a first animal model of cyproheptadine in the treatment of urothelial carcinoma (12). It demonstrated that cyproheptadine shrunk the size of the tumors in the experimental animal.

## Conclusion

In our previous studies, we had demonstrated that adding cyproheptadine to treatments involving sorafenib significantly improves both the overall survival and progression-free survival of advanced HCC more than treatments that utilize sorafenib alone do (7). For patients in the advanced stage of HCC, we usually suggest a prescription of cyproheptadine in addition to that of sorafenib. However, in this case study, the patient has claimed to recover from HCC with the application of cyproheptadine monotherapy before she was recommended to take sorafenib. For a patient who is in the intermediate stage of HCC, we usually suggest the prescription of cyproheptadine after one has received TACE, according to the above-mentioned large cohort study (8).

Because cyproheptadine demonstrates successful treatment of patients in the intermediate and advanced stages of HCC, we further infer that it would be beneficial for patients in the early stage of HCC. After resection or ablation, if patients are diagnosed with early stage of HCC, cyproheptadine can be initiated to reduce tumor recurrence, which is inexpensive, and has fewer side effects. It would be crucial in finding other successful cases of cyproheptadine monotherapy in HCC treatment. However, more studies such as prospectively clinical trials, and ideally randomized trials are needed to evaluate the role of cyproheptadine monotherapy in the treatment of HCC.

## Data Availability Statement

The raw data supporting the conclusions of this article will be made available by the authors, without undue reservation.

## Ethics Statement

Ethical review and approval was not required for the study on human participants in accordance with the local legislation and institutional requirements. The patients/participants provided their written informed consent to participate in this study. Written informed consent was obtained from the individual(s) for the publication of any potentially identifiable images or data included in this article.

## Author Contributions

Y-MF, T-HC, and DB wrote the manuscript. K-SL undertook the pathological diagnosis. M-CH undertook the radiological diagnosis. Y-MF, C-KC, and C-YC carried out the clinical management of the patient. All authors contributed to the article and approved the submitted version.

## Conflict of Interest

The authors declare that the research was conducted in the absence of any commercial or financial relationships that could be construed as a potential conflict of interest.

The handling editor declared a shared affiliation with one of the authors DB at time of review.

## Publisher’s Note

All claims expressed in this article are solely those of the authors and do not necessarily represent those of their affiliated organizations, or those of the publisher, the editors and the reviewers. Any product that may be evaluated in this article, or claim that may be made by its manufacturer, is not guaranteed or endorsed by the publisher.
